# Pembrolizumab on pre-existing inclusion body myositis: a case report

**DOI:** 10.1186/s41927-020-00144-5

**Published:** 2020-09-16

**Authors:** Naohiro Uchio, Atsushi Unuma, Toshiyuki Kakumoto, Masao Osaki, Yoshitaka Zenke, Kenichi Sakuta, Akatsuki Kubota, Yoshikazu Uesaka, Tatsushi Toda, Jun Shimizu

**Affiliations:** 1 Department of Neurology, Graduate School of Medicine, The University of Tokyo, 7-3-1 Hongo, Bunkyo-ku, Tokyo, 113-8655 Japan; 2 Department of Neurology, Toranomon Hospital, 2-2-2 Toranomon, Minato-ku, Tokyo, 105-8470 Japan; 3 Division of Thoracic Oncology, National Cancer Center Hospital East, 6-5-1 Kashiwanoha, Kashiwa-shi, Chiba, 277-8577 Japan; 4 Department of Neurology, Kashiwa Hospital, Jikei University School of Medicine, 163-1 Kashiwashita, Kashiwa-shi, Chiba, 277-8567 Japan; 5 Department of Physical Therapy, Tokyo University of Technology, 5-23-22, Nishikamata, Ota-ku, Tokyo, 144-8535 Japan

**Keywords:** Inclusion body myositis, Immune checkpoint, Pembrolizumab, Immune-related adverse events

## Abstract

**Background:**

Cases of exacerbation of pre-existing neuromuscular diseases induced by immune checkpoint inhibitors (ICIs) have rarely been reported because patients with autoimmune diseases have generally been excluded from ICI therapy due to the increased risk of exacerbation. We describe the first case of an elderly patient who experienced exacerbation of a previously undiagnosed sporadic inclusion body myositis (sIBM), the most common myopathy in the geriatric population, which was triggered by anti-programmed cell death-1 therapy.

**Case presentation:**

A 75-year-old man who was receiving pembrolizumab presented with limb weakness. Three years prior, he had noticed slowly progressive limb weakness, but he received no diagnosis. After the first infusion of pembrolizumab, his creatine kinase (CK) levels had increased. The neurological examination and muscle biopsy findings confirmed the diagnosis of sIBM and suggested exacerbation of sIBM induced by pembrolizumab. After the patient’s CK levels decreased, pembrolizumab was restarted. The tumor progressed after its treatment with pembrolizumab. The patient died after 15 months of follow-up.

**Conclusions:**

In patients with slowly progressive limb weakness, sIBM should be explored before ICI therapy. In addition, if patients show high CK levels after ICI introduction, it is necessary to confirm whether they have sIBM in order to avoid unnecessary immunosuppressive therapies and assess whether they can tolerate ICI reintroduction.

## Background

Immune checkpoint inhibitors (ICIs) potentiate T-cell activity and show dramatic efficacy in treating cancers, but they may also induce immune-related adverse events (irAEs) resembling autoimmune diseases [1–3]. Several reports have documented ICI-induced exacerbation of pre-existing autoimmune diseases [2, 4, 5]. Regarding neuromuscular irAEs, a case with myasthenia gravis [4] and another with interferon-alpha-induced myositis [2] showing ICI-induced exacerbation have been reported. Because of the rarity of ICI introduction in pre-existing autoimmune diseases, whether each ICI type exacerbates particular autoimmune diseases remains unclear. Herein, we report a case of presumed exacerbation of previously undiagnosed sporadic inclusion body myositis (sIBM) in an elderly patient treated with anti-programmed cell death-1 (PD-1) therapy.

## Case presentation

**Fig. 1:**
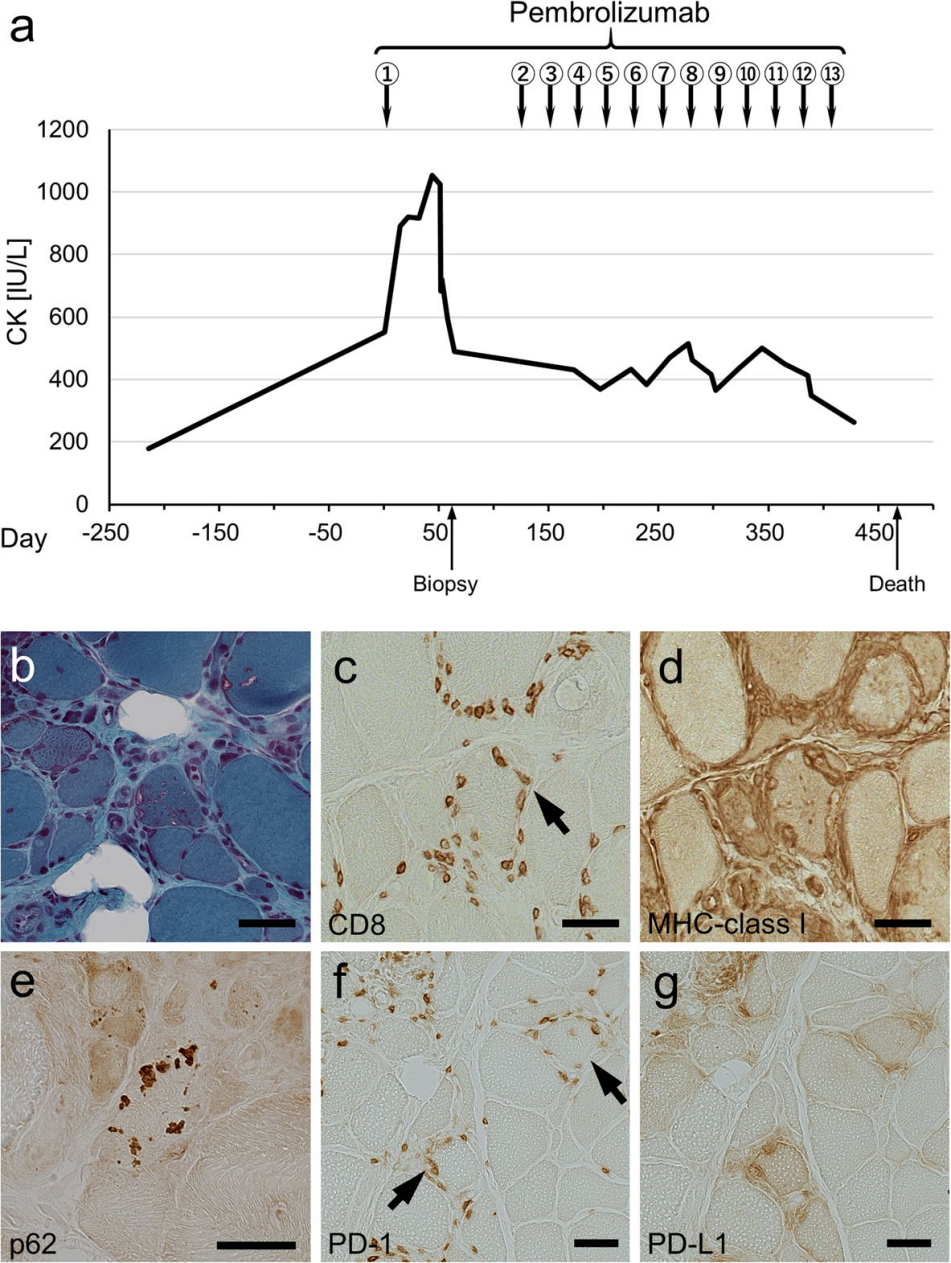
Clinical course and histopathologic features. **a** Time course of serum CK levels. **b**–**g** Histopathological findings of biopsy of the biceps muscle. **b** Gomori trichrome staining showing rimmed vacuoles. **c**, **d** Serial sections of immunohistochemistry for CD8 and MHC class I. **c** Endomysial CD8-positive cells surrounding and invading non-necrotic fibers (arrow). **d** Diffuse expression of MHC class I antigen in non-necrotic fibers. **e** Immunohistochemistry for p62 showing p62-positive cytoplasmic inclusions. **f**, **g** Serial sections of immunohistochemistry for PD-1 and PD-L1. **f** Endomysial PD-1-positive cells surrounding and invading non-necrotic fibers (arrows). **g** PD-L1-positive cells and PD-L1 overexpression in the non-necrotic fibers surrounded by PD-1-positive cells. Bars = 50 μm

A 75-year-old man who received pembrolizumab (a humanized monoclonal anti-PD-1 antibody) for lung squamous cell carcinoma (T2aN3M1b, stage IV) presented with limb weakness. At the age of 72, he showed slowly progressive difficulty in climbing stairs, but his creatine kinase (CK) level was within normal limits 7 months before the pembrolizumab introduction. At the day of the first infusion of pembrolizumab, his CK level was mildly elevated to 552 IU/L, but he showed no additional symptoms. After the first infusion of pembrolizumab, his CK levels increased to 1054 IU/L at day 44 (Fig. 1a). Neurological examination demonstrated diffuse limb weakness including the quadriceps (Medical Research Council [MRC] grade 4/4) and finger flexors (MRC grade 3/3) and atrophy of the quadriceps and paraspinal muscles. Myositis-specific autoantibodies and anti-acetylcholine receptor antibodies were negative. Electromyography indicated an irritable myopathy. Echocardiography showed normal left ventricular function. Muscle biopsy at day 59 demonstrated prominent variation in muscle fiber size, rimmed vacuoles (Fig. 1b), endomysial CD8-positive cell infiltration with invasion of non-necrotic fibers (Fig. 1c), overexpression of major histocompatibility complex (MHC) class I antigen in non-necrotic fibers (Fig. 1d), and p62-positive inclusions (Fig. 1e). Abundant endomysial PD-1-positive cell infiltration fiber (Fig. 1f), programmed death ligand-1 (PD-L1) overexpression in the invaded fibers, and PD-L1-positive cells (Fig. 1g) were also observed. The localization of PD-1-positive cells was consistent with that of CD8-positive cells (data not shown).

The patient was diagnosed with clinicopathologically defined sIBM [6] and suspected exacerbation of sIBM due to anti-PD-1 therapy. Pembrolizumab was discontinued without introduction of immunosuppressive therapy. His CK levels decreased to 489 IU/L at day 64. The patient had been stable disease of his lung cancer after the first infusion of pembrolizumab. However, his brain metastasis was revealed at day 94. After gamma knife radiosurgery for the metastasis, pembrolizumab was restarted at day 122; 12 additional infusions were administered. His limb weakness gradually worsened without further elevation of CK levels. The primary lung lesion progressed after the additional treatment with pembrolizumab and led to the evaluation of progressive disease. The patient died of sepsis, shortly after a 15-month follow-up.

## Discussion and conclusions

The clinicopathologic characteristics of our patient indicated a chronic disease course. Thus, we assumed that he had developed sIBM more than 3 years before receiving the anti-PD-1 therapy. The PD-1/PD-L1 pathway is thought to be involved in the pathogenesis of sIBM [6]. Anti-PD-1 therapy in our patient may have affected the PD-1/PD-L1 pathway, activated CD8- and PD-1-positive T cells targeting the muscle, and finally revealed the existence of sIBM.

Few studies have been conducted on PD-L1 expression in muscle fibers. Wiendl et al. firstly reported that PD-L1 was expressed in inflamed skeletal muscle fibers in polymyositis, dermatomyositis, and sIBM [7]. We also found upregulated PD-L1 expression in non-necrotic skeletal muscle fibers in ICI-induced myositis [1]. Furthermore, PD-L1 expression in inflamed cardiac muscle fibers was reported in ICI-induced cardiomyositis [8]. The significance of PD-L1 expression in muscle fibers is not fully understood, but PD-L1 expression in muscles was proposed to be a protective mechanism to inflammation [1, 7, 8]. Additional studies are needed to elucidate the function of PD-L1 on muscle fibers in inflammation and the effect of ICI therapy on the expression of PD-L1.

In our case, pembrolizumab maintained stable disease for 3 months after the first infusion, suggesting the possible efficacy for his cancer. This finding is consistent with the association between development of irAEs and better responses to ICI and favorable outcomes in patients with lung cancer [9]. More cases should be accumulated to identify factors that may influence patient outcomes for cancers.

Our patient showed no further CK elevation after the second pembrolizumab infusion. Recent papers reported patients with moderate myositis who had no exacerbation after ICI reintroduction [2, 10]. These reported cases and our case suggest that ICI reintroduction does not always cause exacerbation of pre-existing myositis. Notably, a large study on irAEs showed that the recurrence of irAE symptoms after ICI reintroduction is associated with severity and a long duration of initial irAEs [11].

ICI therapy can alter the patient’s immune environment and tumor antigenicity by the upregulation of immune checkpoint molecules, including PD-L1, in a time-dependent manner [12]. In our case, changes in the immune environment due to the ICI administration may have reduced susceptibility to the development of irAE and increased the resistance of the tumor to ICI.

Regarding the judgment of restarting ICI, classifying the severity of an irAE is important, because grade 4 (life-threatening) irAEs are an absolute contraindication for ICI therapy [9]. In our case, the patient’s muscular symptoms were classified as a grade 2 (moderate) myositis, and the highest CK level was also classified as grade 2 (moderate). After confirming that the patient’s CK levels had decreased and noticing his brain metastasis, the doctor in charge decided to resume pembrolizumab for the following reasons: (i) the first infusion showed the potential efficacy in treating his tumor; (ii) the patient was unaware of the chronic symptoms of sIBM; and (iii) the patient strongly desired to continue the therapy. It is reasonable that the decision to restart ICI be judged based on multiple factors that affect each patient individually in the clinical setting.

Guidelines for ICI therapy recommend general pre-therapy work-up to predict irAEs, including physical examination, exploration of pre-existing autoimmune diseases and baseline laboratory and imaging studies [13]. sIBM, the most common and chronic myopathy in the elderly, is often overlooked by patients and unacknowledged by non-neurologists. Considering the increasing opportunities for ICI therapy with elderly patients, we believe that sIBM should be added to the list of autoimmune diseases needed to be explored before ICI therapy. Furthermore, once patients develop irAEs with high CK levels, it is necessary to confirm whether they have sIBM to avoid unnecessary immunosuppressive therapies and to assess whether the patients can tolerate ICI reintroduction.

## Data Availability

The data and materials used and/or analyzed during the current study are available from the corresponding author on reasonable request.

## Abbreviations

CK: Creatine kinase
ICIs: Immune checkpoint inhibitors
irAEs: Immune-related adverse events
MHC: Major histocompatibility complex
MRC: Medical research council
PD-1: Programmed cell death-1
PD-L1: Programmed death ligand-1
sIBM: Sporadic inclusion body myositis
